# Constructing tissue-specific transcriptional regulatory networks via a Markov random field

**DOI:** 10.1186/s12864-018-5277-6

**Published:** 2018-12-31

**Authors:** Shining Ma, Tao Jiang, Rui Jiang

**Affiliations:** 1 Ministry of Education Key Laboratory of Bioinformatics; Bioinformatics Division, Beijing National Research Center for Information Science and Technology; Department of Automation, Tsinghua University, Beijing, 100084 China; 2 Department of Statistics, Department of Biomedical Data Science, Bio-X Program Stanford University, Stanford, CA 94305 USA; 3 Department of Computer Science and Engineering, University of California, Riverside, CA 92521 USA

## Abstract

**Background:**

Recent advances in sequencing technologies have enabled parallel assays of chromatin accessibility and gene expression for major human cell lines. Such innovation provides a great opportunity to decode phenotypic consequences of genetic variation via the construction of predictive gene regulatory network models. However, there still lacks a computational method to systematically integrate chromatin accessibility information with gene expression data to recover complicated regulatory relationships between genes in a tissue-specific manner.

**Results:**

We propose a Markov random field (MRF) model for constructing tissue-specific transcriptional regulatory networks via integrative analysis of DNase-seq and RNA-seq data. Our method, named CSNets (cell-line specific regulatory networks), first infers regulatory networks for individual cell lines using chromatin accessibility information, and then fine-tunes these networks using the MRF based on pairwise similarity between cell lines derived from gene expression data. Using this method, we constructed regulatory networks specific to 110 human cell lines and 13 major tissues with the use of ENCODE data. We demonstrated the high quality of these networks via comprehensive statistical analysis based on ChIP-seq profiles, functional annotations, taxonomic analysis, and literature surveys. We further applied these networks to analyze GWAS data of Crohn’s disease and prostate cancer. Results were either consistent with the literature or provided biological insights into regulatory mechanisms of these two complex diseases. The website of CSNets is freely available at http://bioinfo.au.tsinghua.edu.cn/jianglab/CSNETS/.

**Conclusions:**

CSNets demonstrated the power of joint analysis on epigenomic and transcriptomic data towards the accurate construction of gene regulatory network. Our work provides not only a useful resource of regulatory networks to the community, but also valuable experiences in methodology development for multi-omics data integration.

**Electronic supplementary material:**

The online version of this article (10.1186/s12864-018-5277-6) contains supplementary material, which is available to authorized users.

## Background

The complicated process of transcription in eukaryotes largely attributes to the collaboration among DNA regulatory elements, RNA polymerases, mediator and cohesion complexes, and sequence-specific transcription factors (TFs). Such collaboration is encoded in a comprehensive gene regulatory network that determines how the expression of a gene is regulated, what responses a cell would adopt to answer external stimuli, and which phenotypic consequences a genetic variation could result in [1]. In the recent years, gene regulatory networks has been widely applied to answer a variety of questions, including the explanation of gene expression [2], the identification of disease genes in genome-wide association studies (GWAS) [3, 4], functional annotations for biological pathways [5, 6], and many others [7–9].

A gene regulatory network is often inferred based on high-throughput assays about interactions among transcription factors and their target genes. RNA-seq, as a means of capturing a snapshot of the whole transcriptome, has provided the most abundant data in such studies. For example, Hu and Chen constructed a transcriptional regulatory network in memory CD8+ T cells with gene expression profiles and predicted TF information, and then identified the core TFs [10]. Li et al. constructed a human regulatory regulatory network in glioma with the expression data of TFs and observed the dynamic rewiring of regulators during the glioma progression [11]. Marbach et al. introduced a resource of 394 human gene regulatory networks by integrating TF binding motifs with Cap Analysis of Gene Expression (CAGE) data from the FANTOM5 project [12].

With the promise of detecting TF binding sites at high resolution, ChIP-seq has been used with RNA-seq data to infer gene regulatory networks. For example, Roy et al. constructed a mixed regulatory network that combines transcriptional regulation by TFs from ChIP experiments and posttranscriptional regulation by miRNAs [13]. Chen et al. developed an efficient Bayesian integration method for the inference of regulatory networks using ChIP-seq and RNA-seq profiles [14]. These studies have also suggested that TFs normally bind to their target sites and regulate downstream genes in a cell-type specific manner [15, 16]. Moreover, such specificity is closely related to biological functions and cellular properties [10, 11, 17, 18].

There are several difficulties that restrict large-scale applications of the ChIP-seq technology. Besides the restriction of suitable antibodies for TFs, the number and cost of experiments required by a large number of TFs also limit the feasibility to construct gene regulatory networks for a variety of phenotypes and species via ChIP-seq. To overcome these limitations, DNase-seq has been developed to enable the capture of chromatin accessibility in whole-genome scale [19, 20]. Taking advantage of such merits as free from the consideration of TF-specific antibodies, it has been shown that the regulatory network specific to a cell line can be constructed from a single DNase-seq experiment [21–23]. Moreover, the collection of abundant DNase-seq profiles for major human cell lines in such genomic studies as the ENCODE [24] and Roadmap [25] projects has made the large-scale construction of regulatory networks for a variety of cell lines and tissues possible.

Motivated by the above understanding, we propose in this paper a Markov random field (MRF) model, named CSNets (Cell-line Specific regulatory Networks), that integrates DNase-seq data with RNA-seq data towards large-scale inference of gene regulatory networks. In this method, we first roughly infer regulatory networks for individual cell lines using DNase-seq data alone. Then, we fine-tune these networks using an MRF model, based on pairwise similarity between cell lines derived from RNA-seq data. Focusing on data released by the ENCOODE project, we constructed regulatory networks specific to 110 cell lines and 13 major tissues for human. Using ChIP-seq experimental data as a gold standard, we showed the superior quality of our networks over that obtained by existing methods. Through functional enrichment analysis, we demonstrated that TFs and their predicted targets tend to share similar biological functions. Besides, integrative analysis of our networks with GWAS data of Crohn’s disease and prostate cancer both suggested genes and genetic variants that were either consistent with the literature or provided biological insights into regulatory mechanisms of these two complex diseases.

## Methods

### Data collection

We extracted DNase-seq profiles for 110 human cell lines, representing 70 diverse cell types and 13 unique tissue lineages, from the ENCODE project [26]. We collected gene expression data of corresponding cell lines from the ENCODE project [24]. We derived binding motifs of 368 transcription factors from the JASPAR [27] and TRANSFAC [28] databases. We extracted 353 ChIP-seq experiments from the ENCODE project, corresponding to 108 transcription factors and 59 cell lines. We collected 1454 gene sets with gene ontology (GO) annotations from the MSigDB database [29], involving 233 GO terms of cellular component, 825 terms of biological process, and 396 terms of molecular function.

### Principles of CSNETS

**Fig. 1:**
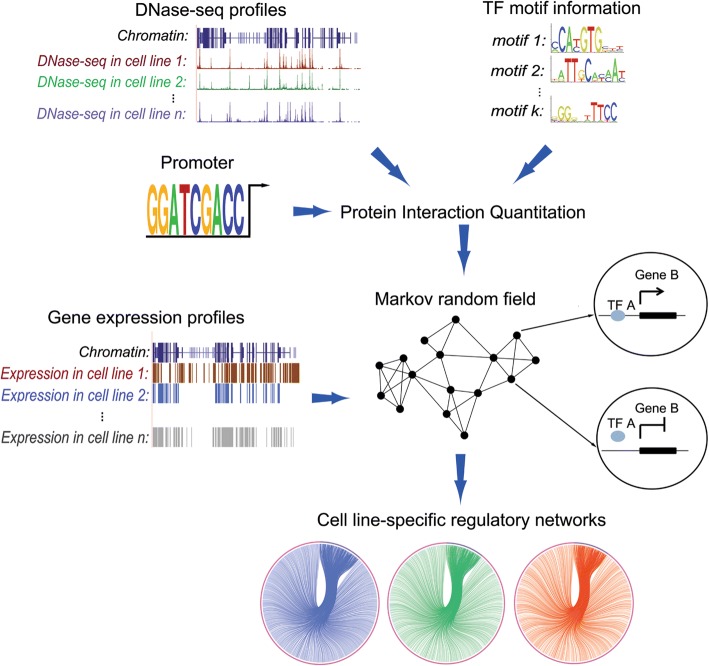
Workflow of the construction of 110 cell line-specific regulatory networks. Firstly, perform PIQ on DNase-seq profiles and TF motifs to predict genome-wide transcription factor binding sites for 368 TFs in 110 cell lines, respectively. Secondly, map these transcription factor binding sites to the promoter regions of genes and thus link TFs to target genes. Thirdly, construct regulatory networks with a Markov random field (MRF) model based on the cell line similarity measured by the expression profiles of these cell lines. Finally, we get the cell line-specific regulatory network for each cell line

We proposed to construct a transcriptional regulatory network specific to a cell line by integrating DNase-seq data, transcription factor binding motif information, and gene expression data, as illustrated in Fig. 1.

We first followed a computational method called Protein Interaction Quantitation (PIQ) [23] to perform a whole-genome prediction of transcription factor binding sites from DNase-seq data. Briefly, PIQ relied on a machine-learning method called expectation propagation [30] to identify binding sites for transcription factors with known motif patterns. Using this method, we obtained the position and binding probability for each predicted binding site, by using DNase-seq data corresponding to the 110 cell lines, the reference sequence of *Homo sapiens* (GRCh37), and position weighted matrix of motif for the 368 transcription factors. Focusing on predicted binding sites in promoter regions (TSS ± 2 kb), we linked transcription factors to their target genes, and thus obtained preliminary regulatory networks specific to the 110 human cell lines. Second, we incorporated gene expression data and adopted a rigorous Markov random field model to fine-tune these preliminary networks. The basic assumption behind this model is that similar cell lines tend to share similar regulatory patterns. With this understanding, we used gene expression data to measure the similarity between cell lines, and then connect regulatory relationships of different cell lines by using a Markov random field (MRF) model based on the similarity. Detailed explanations of components in our method are given below.

### Quantification of cell line similarity

We adopted a measure, called TSI (Tissue Similarity Index) [31], to characterize relationships among cell lines and quantify their degree of similarity. First, we used SAM [32] to identify 592 genes that were differentially expressed (*q*-value = 0) in the 110 cell lines. Then, we applied the singular value decomposition (SVD) to expression data of these genes to perform a dimensionality reduction. In detail, expression values of a gene across the 110 cell lines was first normalized to zero mean and standard deviation one. The resulting expression matrix regarding the 592 genes and 110 cell lines were then decomposed into USV^*T*^, where columns of U were called eigenarrays, diagonals in S singular values, and rows of V^*T*^ right singular vectors. Finally, we characterized the similarity between two cell lines as the Pearson’s correlation coefficient of the first 16 dimensions of the eigenarrays in the SVD decomposition. Here, we calculated CSI values based on different numbers of dimensions and found that they were robust and slightly changed. We name such a similarity measure as the Cell-line Similarity Index (CSI).

### Markov random field model

We proposed a Markov random field model to fine-tune the preliminary networks. Specifically, for a given regulatory relationship (e.g., TF *A* regulates target gene *B*), we constructed an MRF network G = {V_*AB*_, E_*AB*_}, where a node *v*_*ABi*_ ∈ *V*_*AB*_, (*i* = 1, 2, …, 110) indicates the regulation of TF *A* on target gene *B* in cell line *i*, and an edge (*i*, *j*) ∈ *E*_*AB*_ denotes the regulation coherence for TF *A* and gene *B* between cell line *i* and *j*. We introduced an indicator variable *x*_*i*_ ∈ *X* for the node *v*_*ABi*_, indicating whether the regulation exists (*x*_*i*_ = 1) or not (*x*_*i*_ = 0) in cell line *i*. Suppose that the higher the degree of similarity between cell line *i* and *j*, the stronger the positive correlation of variable *x*_*i*_ and *x*_*j*_, we define a criterion called the TF non-specific index (TNI) as the proportion of common targets for the TF involved in cell line *i* and *j*. The larger the TNI value, the more similar of the regulatory mechanism corresponding to the concerned TF. We define the edge weight, *w*_*ij*_ ∈ *W*, as the average of CSI(*x*_*i*_, *x*_*j*_) and TNI(*x*_*i*_, *x*_*j*_). We set a threshold c with default value 0.5, and regarded cell line *i* and *j* as connective if and only if *w*_*ij*_ > c.

We then followed the literature [33] to construct a pairwise MRF model that uses the similarity information between cell lines to assist the prediction on the existence of a transcriptional regulatory relationship in the cell lines. This model contains two types of potential functions. The first type is called the node function, defined as

$$ {\phi}_i\left({x}_i\right)=\left\{\begin{array}{c}{P}_{\left(1,i\right)}/{P}_{\left(0,i\right)}\ \mathrm{if}\ {P}_{\left(1,i\right)}>{P}_{\left(0,i\right)},{x}_i=1\ \\ {}{P}_{\left(0,i\right)}/{P}_{\left(1,i\right)}\ \mathrm{if}\ {P}_{\left(0,i\right)}>{P}_{\left(1,i\right)},{x}_i=0\\ {}1,\mathrm{otherwise}\end{array}\right. $$

where *P*_(1, *i*)_ and *P*_(0, *i*)_ denote the probability of existence (*x*_*i*_ = 1) or failure (*x*_*i*_ = 0) of the given regulatory relationship in cell line i, respectively. We used the probability of TF binding inferred from PIQ as the probability of *P*_(1, *i*)_.

The second type of potential function is called the edge function, defined as

$$ {\psi}_{\left(i,j\right)}\left({x}_i,{x}_j\right)=\left\{\begin{array}{c}{e}^{\left(\mathrm{CSI}\left({x}_i,{x}_j\right)+\mathrm{TNI}\left({x}_i,{x}_j\right)\right)/2},\mathrm{if}\ {x}_i={x}_j,\\ {}1,\mathrm{otherwise}.\end{array}\right. $$

This function uses the CSI and TNI score mentioned above to measure the association between cell lines.

With the definition of two types of potential functions, the joint distribution of all indicator variables X can be denoted as

$$ \Pr (X)=\frac{1}{Z}\prod \limits_{\left(i,j\right)\in {E}_{AB}}{\psi}_{\left(i,j\right)}\left({x}_i,{x}_j\right)\prod \limits_{i=1}^n{\phi}_i\left({x}_i\right) $$

where *Z* represents the partition function, making the sum of the probabilities equal to 1. Through a negative logarithmic transformation, the joint distribution of X can be written as

$$ E(X)=-\gamma -\sum \limits_{i=1}^n\ln {\phi}_i\left({x}_i\right)-\sum \limits_{\left(i,j\right)\in {E}_{AB}}\ln {\psi}_{\left(i,j\right)}\left({x}_i,{x}_j\right) $$

where *γ* is a constant. *E*(*X*) is named as pseudo-energy function. With this formulation, we transformed the problem of maximizing the joint distribution Pr(*X*) into that of minimizing the pseudo-energy function [33, 34]. We then applied iterated conditional modes [35] to further transform the problem of minimizing the pseudo-energy function into the maximum flow problem of networks.

In detail, firstly we define *α*_*i*_(*x*_*i*_) = ln *ϕ*_*i*_(*x*_*i*_)and *β*_*ij*_(*x*_*i*_, *x*_*j*_) = ln *ψ*_(*i*, *j*)_(*x*_*i*_, *x*_*j*_). When the value of *x*_*i*_ is not consistent with its probability distribution (i.e. *P*_(0, *i*)_ > *P*_(1, *i*)_ when*x*_*i*_ = 1 or *P*_(0, *i*)_ < *P*_(1, *i*)_ when*x*_*i*_ = 0), the value of *α*_*i*_(*x*_*i*_) is 0, rather than ∣ ln *ϕ*_*i*_(1) − ln *ϕ*_*i*_(0)∣. When cell lines *i* and *j* are connective and *x*_*i*_ and *x*_*j*_ are of differential values, the value of *β*_*ij*_(*x*_*i*_, *x*_*j*_) is 0 rather than (CSI(*x*_*i*_*, x*_*j*_) + TNI(*x*_*i*_*, x*_*j*_))/2. It is verified that we can transform the problem of minimizing the pseudo-energy function into that of summing total losses of *α*_*i*_(*x*_*i*_)and *β*_*ij*_(*x*_*i*_, *x*_*j*_) when the following inequality satisfies [36].

$$ {\beta}_{ij}\left({x}_i=1,{x}_j=1\right)+{\beta}_{ij}\left({x}_i=0,{x}_j=0\right)\ge {\beta}_{ij}\left({x}_i=1,{x}_j=0\right)+{\beta}_{ij}\left({x}_i=0,{x}_j=1\right) $$

This equation suggests that the problem of minimizing the pseudo-energy function is transferred into the maximum flow problem of networks. We finally applied the loopy belief propagation algorithm [37] to calculate the probability distribution of X.

### Evaluation using ChIP-seq data

We collected 353 ChIP-seq experiments, regarding 108 TFs in 59 cell lines, from the ENCODE project. We then evaluated the contribution of the MRF model as follows.

We first generated a gold standard of target genes for a TF in a cell line from the corresponding ChIP-seq experiment. To achieve this objective, we mapped binding sites identified in the experiment to promoter regions (TSS ± 2Kbps) of protein coding genes and assigned experimental scores of the binding sites to the mapped genes, which were used as candidate target genes. To further reduce false positives in these genes, we identified the median size (*M*) of three gene sets, which include candidate target genes according to ChIP-seq data, target genes of the TF according to the network constructed by the MRF model for the given cell line, and target genes of the TF according to the preliminary network for the given cell line. Finally, we ranked candidate target genes according to their scores and used those ranked among top *M* as the gold standard of target genes for the TF in the given cell line.

We then performed a ROC analysis to evaluate the quality of the networks constructed by our method. Given a TF and a cell line, we used target genes identified by the corresponding ChIP-seq experiment as the positive set, and the reset genes as the negative set. Focusing on the list of target genes for a TF given by our method, at a cut-off value of the regulatory probability, we calculated the sensitivity as the proportion of positives whose regulatory probability is higher than the cut-off, and the specificity as the proportion of negatives whose regulatory probability is lower than the cut-off. Varying the cut-off value, we drew a receiver operating characteristic (ROC) curve (sensitivity versus 1-specificity) and calculated the AUC score as the area under this curve. In a similar way, we obtain the AUC score of for the preliminary network. The relative change of these two AUC scores is then used to compare the performance of these two networks, for the given TF in the give cell line.

We further evaluated quality of the constructed networks by checking the overlap between target genes in the networks and those identified by ChIP-seq experiments. This was done by filtering out low confidence target genes that were ranked below the threshold *M* and then counting the number of genes shared in the remaining target genes. Using a similar strategy, we obtained an overlapping score for the preliminary network. The relative change of these two scores can then be used to compare the performance of these two networks, for the given TF in the give cell line.

## Results

### Regulatory networks specific to 110 cell lines and 13 tissues

We constructed regulatory networks specific to 110 human cell lines, and we further merged networks specific to cell lines belonging to the same tissues to obtain 13 tissue-specific regulatory networks, as summarized in Table 1. From the table, we observe that the network specific to stem cell has the largest in-degree and out-degree. This phenomenon can probably be explained by the pluripotency nature of stem cells. We also notice that the liver-specific network also has high degrees.

**Table 1:** Property for 13 tissue-specific networks

Tissue	Node	Edge	In-degree	Out-degree
Epithelial	19,535	388,433	19.98	1063.34
Fibroblast	19,345	385,268	20.02	1051.29
Muscle	19,349	386,523	20.08	1056.50
Brain	19,602	443,826	22.55	1268.87
Hematopoietic	19,316	452,915	23.44	1277.08
Primitive	19,369	377,249	19.57	1083.85
Skin	19,430	380,265	19.65	1088.57
Stem	20,803	628,780	30.02	1735.75
Endothelial	19,319	454,054	23.62	1234.11
Cervix	19,830	495,002	25.09	1404.84
Liver	19,354	486,529	25.17	1437.27
Prostate	19,146	431,096	22.52	1171.46
Mammary	20,019	496,650	24.91	1380.05

**Fig. 2:**
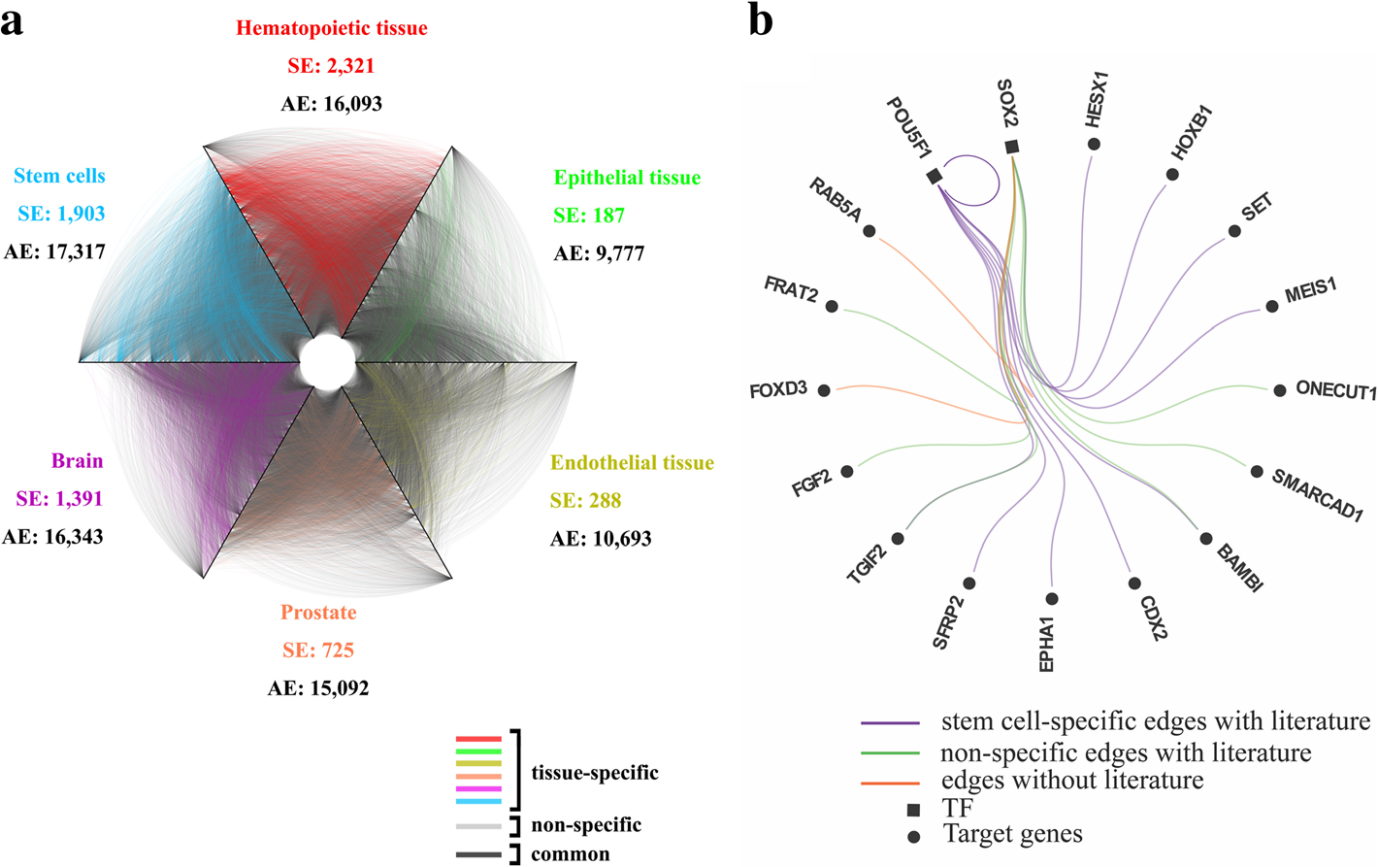
**a** TF-TF tissue-specific regulatory networks and (**b**) core transcriptional regulatory network in human embryonic stem cells. SE means specific edges and AE means all edges

We then extracted sub-networks regarding transcription factors only from the 13 networks and illustrated 6 of such networks in Fig. 2(a). In comparison with other tissues, tissue-specific regulatory relationships in the hematopoietic tissue and stem cells tend to present more frequently, indicating their high degree of the tissue-specificity. In stem cells, we collected TFs and genes closely correlated with the pluripotency from literature [38, 39] and further analyzed the regulatory interactions among them in Fig. 2(b). We notice that most of the regulatory interactions derived from the network specific to stem cell are testified by literature [38, 39] (shown in purple and green) and present a high degree of the tissue specificity (shown in purple), indicating that regulatory relationships specific to stem cells are highly correlated with the property of pluripotency.

### Constructed networks are consistent with ChIP-seq data

We evaluated contributions of the MRF model using 353 ChIP-seq experiments collected from the ENCODE project. Briefly, we first identified a gold standard of target genes for a TF in a cell line from the corresponding ChIP-seq experiment. Then, we evaluated the improvement of a network constructed by using the MRF model over the corresponding preliminary one in terms of relative changes in the AUC score and the overlapping score, as detailed in Methods.

**Fig. 3:**
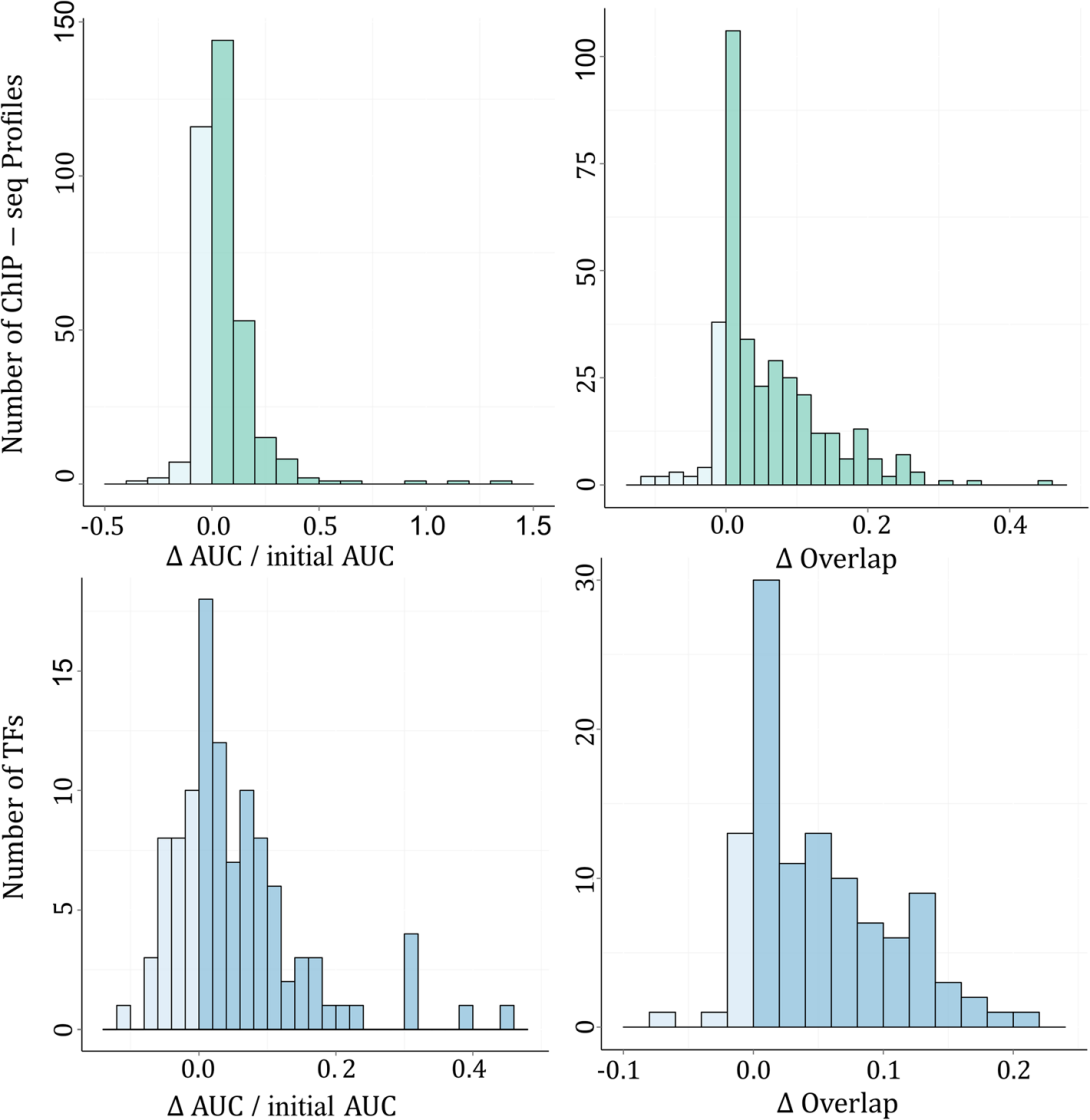
Comparison of the consistency between preliminary/MRF-based networks and ChIP-seq profiles

As shown in Fig. 3(a), the MRF model improves the accuracy in recovering true target genes for a TF in a specific cell line, according to criterion of the relative change in AUC scores. In 225 out of the 353 (64%) experiments, networks constructed by using the MRF model show higher consistency with ChIP-seq data than the preliminary network. Such positive contribution of the MRF model is further supported by the one-sided binomial exact test (*p*-value = 8.4e-08). In terms of the relative change in the overlapping score, as shown in Fig. 3(b), networks constructed by using the MRF model show higher consistency with ChIP-seq data in 287 out of the 353 (81.3%) experiments. The positive contribution of the MRF model is again supported by the one-sided binomial exact test (*p*-value< 2.2e-16).

We further performed a TF level evaluation by aggregating ChIP-seq experiments according to TFs and averaging a criterion over corresponding cell lines. As shown in Fig. 3(c), the MRF model shows higher consistency with ChIP-seq data for 78 out of the 108 (72%) TFs, and the positive contribution of the MRF model is supported by the one one-sided binomial exact test (*p*-value = 1.1e-05). In terms of the relative change in the overlapping score, networks constructed by using the MRF model show higher consistency with ChIP-seq data for 88 out of the 108 (81.5%) TFs. Again, he positive contribution of the MRF model is supported by the one-sided binomial exact test (*p*-value< 6.4e-15).

### Constructed networks are consistent with taxonomy

We testified the rationality of the tissue similarity measured by gene expression data and observed the consistency between regulatory networks and the human cell hierarchical taxonomy graph. After extracting the directed acyclic subgraph of the human cell hierarchical taxonomy graph from the Foundational Model of Anatomy Database [40] in the Unified Medical Language System [41] (shown in Additional file 1: Figure S1), we performed the hierarchical clustering on tissues and cell lines based on gene expression and regulatory relationships respectively, and compare them with the human taxonomy graph.

**Fig. 4:**
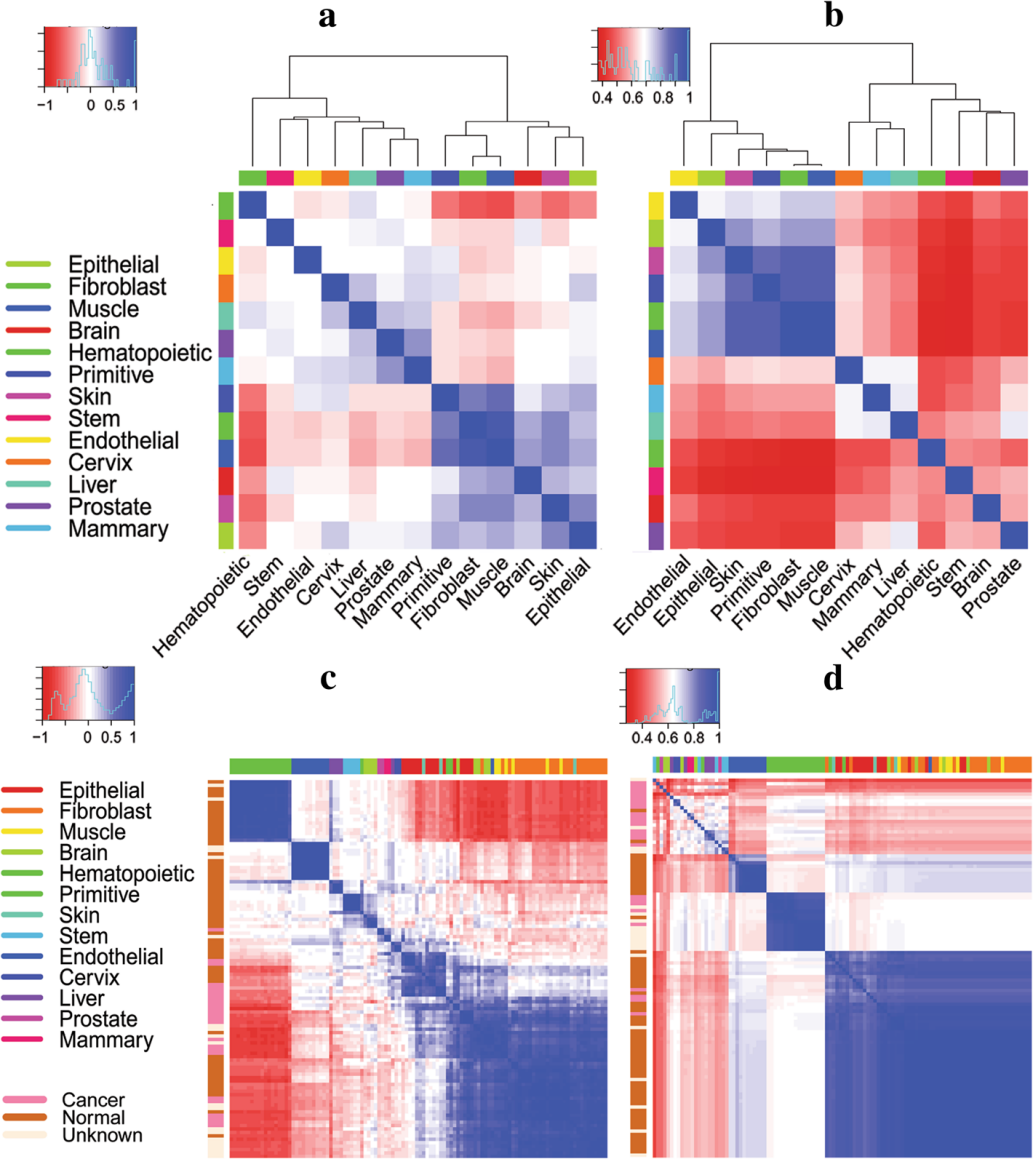
Hierarchical clustering of tissues ((**a**), (**b**)) and cell lines ((**c**), (**d**)) based on expression profiles ((**a**), (**c**)) and regulatory networks ((**b**), (**d**))

First, we performed hierarchical clustering of tissues according to gene expression profiles. Figure 4(a) shows that the hematopoietic tissue is the most distal to the other tissues. The fibroblast and muscle are clustered together, and the prostate tissue is in short distance with the liver. The endothelial tissue and cervix are very close. The epithelial tissue is the parent node of the skin and brain in the human cell hierarchical taxonomy graph, and they are clustered together as well. Therefore, we conclude that it is reasonable to measure the tissue similarity based on gene expression profiles.

Further, hierarchical clustering was performed for the 110 cell lines based on their similarity index, with results shown in Fig. 4(c). We find that cell lines from the same tissues tend to be clustered together, indicating that such cell lines in general have higher similarity than those from different tissues. Moreover, in the 110 cell lines, there are 25 cancer cell lines, 65 cell lines with normal karyotype and 20 unidentified cell lines. Cell lines with the same karyotype are more likely to be in the same cluster.

Next, we inspected the consistency between regulatory networks and the human cell hierarchical taxonomy graph to evaluate the rationality of the tissue similarity measured by regulatory networks. For this objective, we merged regulatory networks specific to cell lines belonging to the same tissue, used the Jaccard coefficient of tissue-specific regulatory networks to measure the similarity between tissues, and then perform hierarchical clustering on the 13 tissues, as shown in Fig. 4(b). The skin, fibroblast, muscle, endothelial and epithelial tissues are clustered together, of which fibroblast and muscle are the closest. The epithelial tissue is close with skin, and these four tissues are relatively distant from the endothelial tissue. The hematopoietic tissue is the most distal one from the others, followed by stem cells. These results are consistent with the human cell hierarchical taxonomy graph. We assert that our regulatory networks describe explainable tissue similarity relationships.

Finally, we performed hierarchical clustering on the 110 cell lines based on their specific regulatory networks. The results, in Fig. 4(d) shows that cell lines from the hematopoietic and endothelial tissues are clustered, respectively, and those from the skin, fibroblast, muscle, endothelial and epithelial tissues are clustered together, which is consistent with the human cell hierarchical taxonomy graph. Similar to the observation from the results of expression profiles, cell lines of the same karyotype are more likely to be closely clustered.

### Correlation between expression of TFs and target genes

**Fig. 5:**
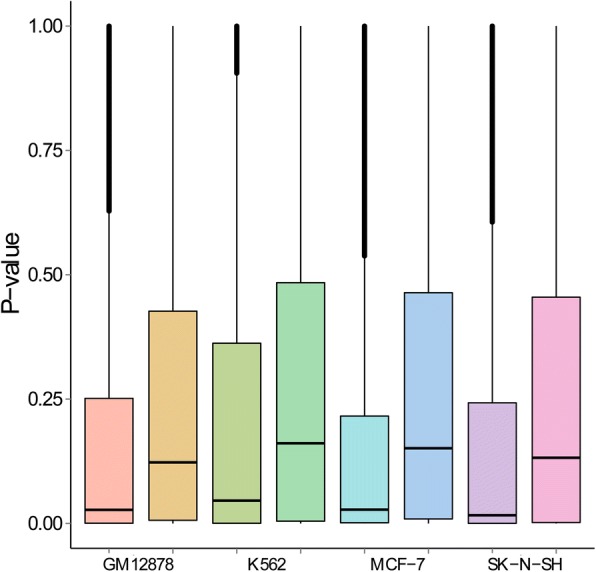
The correlation of expression for TF-gene pairs that are included and not included in the four cell line-specific regulatory networks

We evaluated whether expression of TFs exhibited positive correlation with their predicted target genes in a cell line-specific manner. For this objective, we collected gene expression data for the GM12878, K562, MCF-7, and SK-N-SH cell lines. For each of these cell lines, we calculated Pearson’s correlation coefficient between a TF and each of its target genes, and we plot the statistical significance of the correlation coefficient in Fig. 5. From the figure, we clearly see that TFs show stronger correlation with their target genes in networks constructed by using the MRF model, and one-sided Wilcoxon tests support this observation (*p*-values < 2.2e-16 for all these cell lines). These results suggest that the networks constructed by using our method can well characterize the regulatory relationship between transcription factors and their target genes.

### GO enrichment analysis

With the assumption that a TF and its target genes tend to share common biological functions, we performed a functional enrichment analysis on the target genes of a TF. To achieve this objective, we collected 1454 gene sets from the MSigDB database, covering 825 biological process terms, 396 molecular function terms, and 233 cellular component terms in GO. We then derived two criteria to characterize the degree that a TF and its target genes share a common function, as follows.

Given a TF, a cell line and a GO term, we identified target genes of the TF in the network specific to the cell line, and we performed a Fisher’s exact test to see whether the target genes were enriched in the function corresponding to the GO term. With results of such an analysis for every TF, in every cell line, and for every GO term collected, we were able to derive a statistic to indicate consistency between functions of TFs and their target genes. Particularly, we defined such a statistic as the proportion of significant tests (FDR ≤ 0.2) over all tests performed, and we calculated a statistic for each of the three GO categories, biological process, molecular function, and cellular component, separately. Because all the 1454 GO terms were used in the above analysis, we referred to such a statistic as the total enrichment score. By contrast, we repeated the above enrichment analysis with GO terms not relevant to a TF discarded, and we referred to this formulation as positive enrichment analysis.

**Fig. 6:**
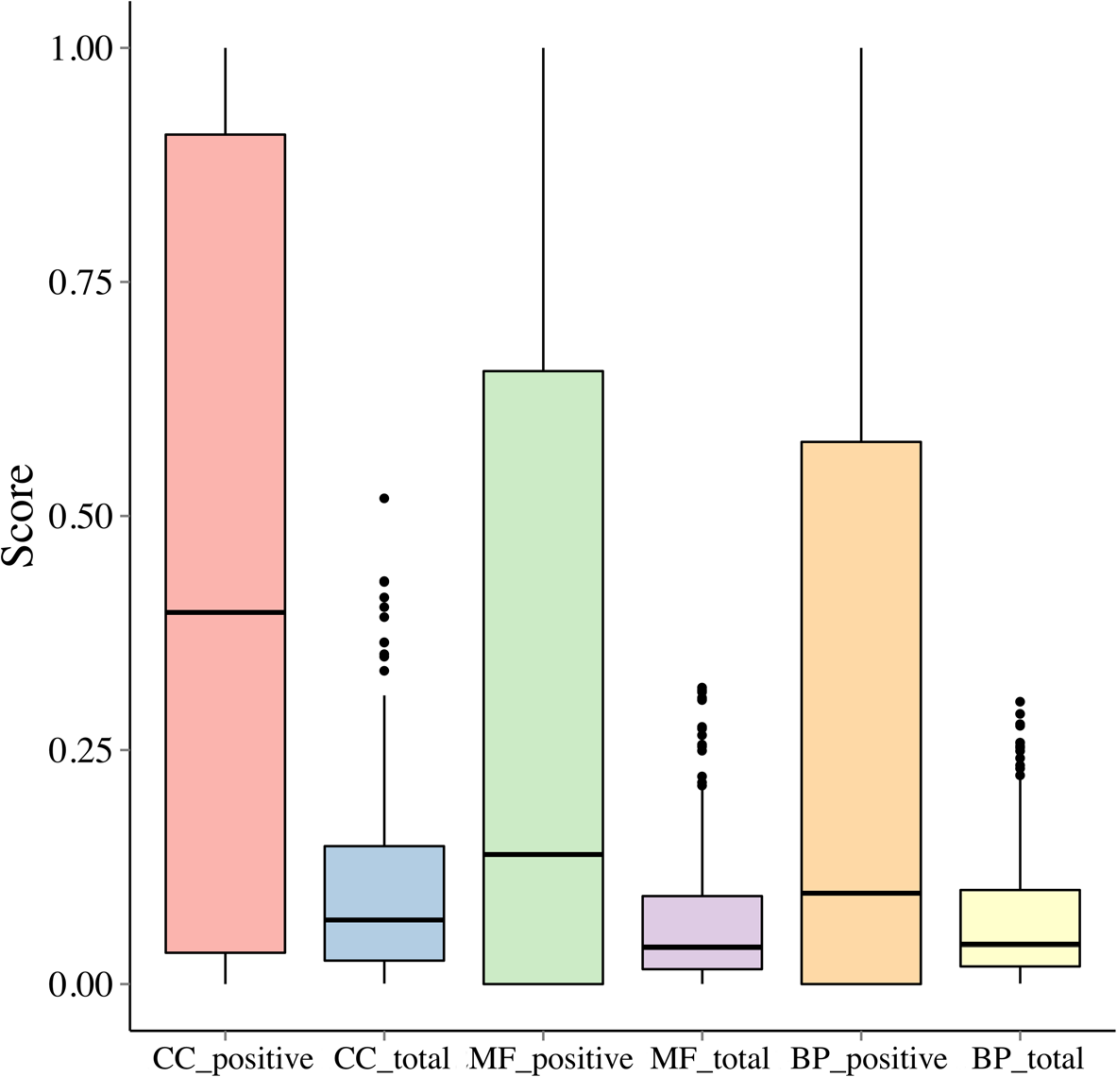
Enrichment results for positive/entire GO terms of three categories

As shown in Fig. 6, it is clear that for each the three GO categories, the positive enrichment analysis exhibits much higher score than the total enrichment analysis, suggesting that TFs indeed tend to have similar functions as their target genes. One-sided Wilcoxon rank sum test further support this conclusion, in that *p*-values are as small as 5.60 × 10^− 7^, 7.95 × 10^− 3^ and 7.80 × 10^−4^for the cellular component, biological process and molecular function, respectively.

### Identification of TFs with differential regulation

We further analyzed whether target genes of a TF exhibit different functions in different cell lines, especially in normal and cancer cell lines. To achieve this objective, we identified 65 normal cell lines and 25 cancer cell lines. For each TF, we collected its target genes, and we performed functional enrichment analysis to see whether functions of the TF were enriched in its target genes, for the normal and cancer cell lines, separately. We showed TFs and GO terms with most significant enrichment *p*-values in Additional file 1: Table S1, from which we can see that many of these TFs have been verified to associate closely with various types of cancer.

**Fig. 7:**
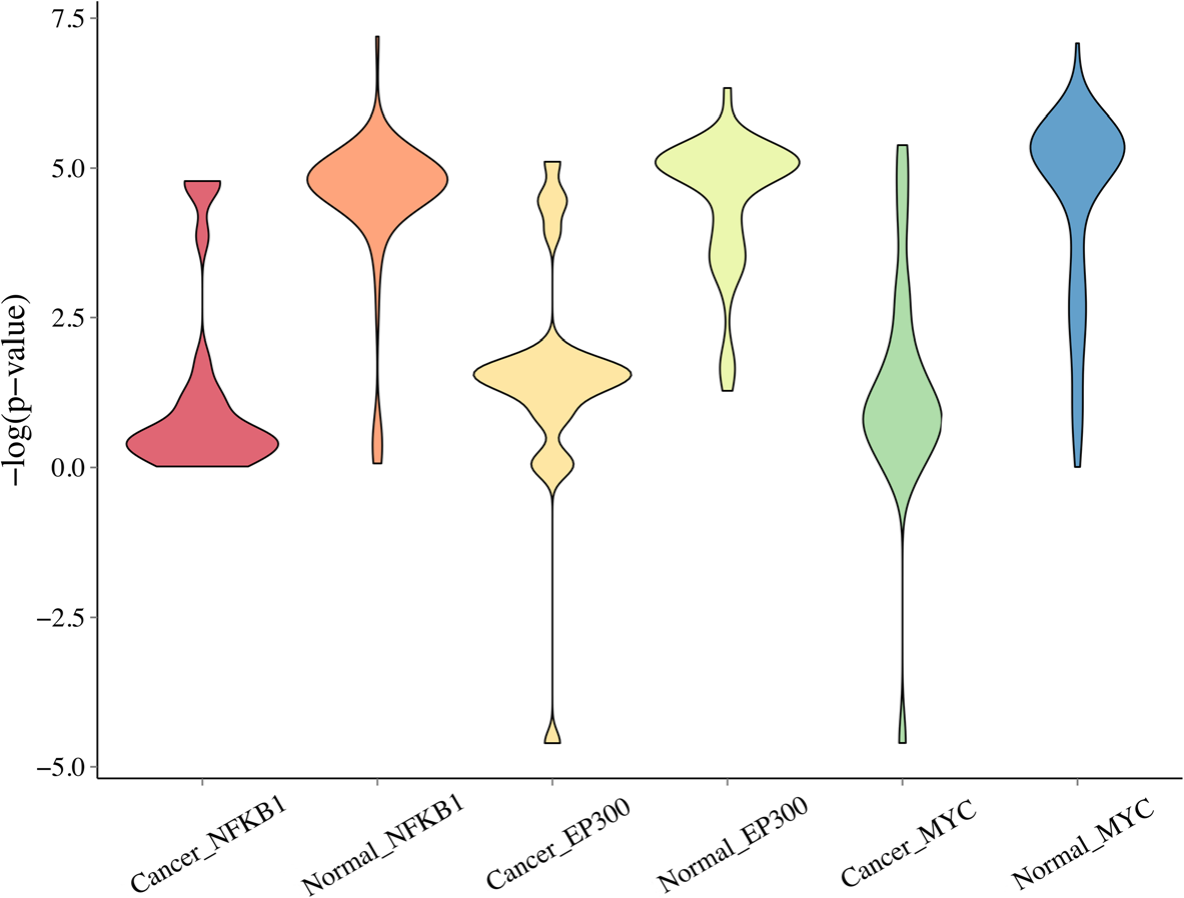
The enrichment results of NFKB1, EP300 and MYC in their corresponding GO terms

For example, EP300 plays an important role in regulating cell growth and blocking the promotion of cancerous tumors. The targets of EP300 are enriched in normal cells rather than cancer cells in two GO terms, corresponding to apoptotic process and programmed cell death respectively, indicating the function of EP300 is altered in cancer cells (Fig. 7). As for FOSL1, the enrichment degree of its targets in the cell proliferation term is significantly different between normal and cancer cell lines. Therefore, it is reasonable to presume that the regulatory mechanisms of these TFs are perturbed in various cancer cells and further affect the growth and promotion of cancers through the matched GO annotated functions.

### Locating TFs for Crohn’s disease

We applied the constructed regulatory networks to analyze a GWAS data set of Crohn’s disease, demonstrating the potential of these networks in identifying disease-related TFs and their regulatory mechanisms. We first select a regulatory network that is specific to Crohn’s disease from the 110 networks. On one hand, it is generally thought that the inflammatory reaction in Crohn’s disease is driven by the activated type 1 helper T cells (Th1) [42]. On the other hand, from the 1000 Genomes Project [43], we observed that the similarity between Th2 cells and Crohn’s disease is the highest among all ENCODE cell lines (Additional file 1: Figure S2). A comparative study on the regulatory networks of these two cell lines shows that these networks share 98.6% edges. We therefore selected the regulatory network of Th1 cell line according to the literature.

We then collected GWAS data for this disease from the literature [44] and calculated for each gene a *p*-value that indicates the strength of association between the gene and this disease, using the tool Pascal [45]. By ranking genes based on their *p*-values, we obtained a gene list, in which top ranked genes can be treated as candidate disease genes. To avoid determining true disease genes based on a hard cut-off of the *p*-value, we resorted to GSEA [46] to assign an enrichment score to a TF, measuring whether its target genes are enriched in highly ranked candidate genes. We further ranked TFs according to their enrichment scores and obtained a list of 114 TFs, in which top-ranked TFs are considered as relevant to Crohn’s disease. From the ranking list, we find that TFs of the NFκB family are relatively ranked high, with NFKB1 ranked first (Table 2). Previous studies [47–50] have shown that the NFκB family is activated and plays a key role in the inflammatory bowel diseases, especially Crohn’s disease.

**Table 2:** The rank results of the NFκB family

TF	*p*-value from GSEA	Rank
NFKB1	2.13E-05	1
REL	3.50E-03	6
NFKB	2.53E-02	17
RELA	1.10E-01	31

We further explored the importance of the cell line specificity of a regulatory network in identifying the NFκB family in the above analysis. Briefly, we extracted target genes of the NFκB family for each of the 110 networks and used Fisher’s exact test to measure the degree of enrichment of these target sets in the candidate genes identified above for Crohn’s disease. Results show that the target gene set of the NFκB family in Th1 cell line is ranked 2nd, and specifically, the target gene set of NFKB1 in Th1 cell line is ranked 1st, among the 110 cell lines (Additional file 1: Figure S3). We therefore conclude that the cell line-specificity of regulatory networks plays an important role in detecting disease-associated TFs.

To gain understanding about the mechanism of Crohn’s disease, we further investigated target genes of the NFκB family in the Th1 cell line and surveyed their correlation with Crohn’s disease according to literature. We found that Franke et al. confirmed 71 distinct loci for Crohn’s disease and listed functionally interesting candidate genes [44]. We then listed the target genes supported by this literature in Table 3. We suspected that the mutations near these functional candidate genes may affect the binding affinity of the NFκB family and hence implicate in Crohn’s disease pathogenesis.

**Table 3:** Targets of the NFκB family correlated with Crohn’s a supported by literature

TF	Target gene	dbSNP ID	Chr.	Risk allele
RELA	PTGER4	rs11742570	5p13	C
NFKB1/RELA	IRF1	rs12521868	5q31	T
NFKB1	SNAPC4	rs4077515	9q34	T
RELA	JAK2	rs10758669	9p24	C
REL/RELA	SMAD3	rs17293632	15q22	T
NFKB1/REL/RELA	GPX4	rs740495	19p13	G
REL/RELA	ICAM1	rs12720356	19p13	G
NFKB1	CREM	rs12242110	10p11	G
NFKB1/RELA	MUC1	rs3180018	1q22	A
NFKB1/REL/RELA	SCAMP3	rs3180018	1q22	A
NFKB1	ZPBP2	rs2872507	17q21	A
REL	FADS1	rs102275	11q12	C

### Differential regulation analysis on the LNCaP cell line

We studied whether an environmental stimulus would affect the regulatory network related to a phenotype by using androgen-treated and untreated LNCaP cells as an example. To achieve this objective, we first compared regulatory networks specific to these two cell lines and found the Jaccard index between edges of these two networks is 0.703. We then focused on edges differently presented in these two networks to obtain a differentially network and performed the following analysis.

We ranked TFs based on the number of their target genes in the differential network in descending order and showed top 5 TFs in Additional file 1: Table S2. In this list, we found that SREBF1 and TWIST are androgen responsive in human cells, and SREBF1 and NFATC2 are androgen responsive in mouse cells, according to the Androgen Responsive Gene Database [51].

We collected target genes for TFs in treated and untreated LNCaP cell lines, respectively, and calculated a Jaccard distance to indicate the proportion of differential regulating edges for a TF. The top 5 TFs with the highest Jaccard distance are present in Table 4. In this list, NFIX is verified to be androgen responsive in human cells, and NFIX, NFATC2 and FOSL1 are verified in mouse cells by the Androgen Responsive Gene Database.

**Table 4:** Top-ranked TFs based on the Jaccard distance

TF	No. of differentially regulating edges	Jaccard distance	Rank
NFIX	3103	0.987902	1
NFATC2	6825	0.98004	2
BATF	2261	0.954814	3
FOSL1	1726	0.953591	4
HIVEP2	1697	0.920282	5

We finally ranked the target genes in the differential regulatory network according to the number of edges pointing to them, say, the number of TFs differentially regulating these genes. The top 5 genes having the largest number of regulation are shown in Table 5. In this list, WWTR1 is known as a downstream regulatory target in the Hippo signaling pathway that plays a key role in tumor suppression. CDKN1A encodes protein *p*21, which plays an important role in KEGG [52] prostate cancer pathway and is again verified by the Androgen Responsive Gene Database.

**Table 5:** Top-ranked genes based on their differential regulated edges

Gene	No. of differentially regulated edges	Rank
WWTR1	41	1
WWTR1-AS1	40	2
CDKN1A	39	3
PRRC2C	38	4
UBE2D3	38	4

## Conclusions and discussion

In this paper, we have proposed a Markov random field model for integrating chromatin accessibility and gene expression data to construct regulatory networks specific to 110 cell lines and 13 tissues. We have demonstrated the high quality of these networks via comprehensive statistical analysis based on ChIP-seq experiments, functional annotations, taxonomic analysis, and literature surveys. Joint analysis of these networks with GWAS data provides results that are either consistent with literature or provided biological insights into regulatory mechanisms of human inherited diseases.

Main contributions of our work include the following aspects. First, we demonstrated the power of joint analysis on epigenomic and transcriptomic data towards the accurate construction of gene regulatory network. In the recent years, parallel assays of the epigenome and transcriptome has become popular, and computational methods for integrative analysis of such data are of urgent need, especially in single-cell multi-omics data analysis [53, 54]. Our work, as a beneficial attempt in this direction, can thus provide valuable experiences in methodology development for multi-omics data integration.

Second, our work provides a useful resource of regulatory networks to the community. Recently, Marbach et al. constructed 394 gene regulatory networks specific to human cell types and tissues by integrating TF motifs with CAGE data from the FANTOM5 project [12]. We compared their results with our networks on four shared cell lines involving 220 common TFs and found the proportion of shared edges ranging from 26 to 31%. On one hand, our regulatory networks consist with the networks constructed with CAGE data to some extent, indicating the rationality and robustness of our networks. On the other hand, the difference between our networks and theirs shows the complementarity and diversity of these two resources. In this sense, combined use of both resources may offer a complete landscape of human transcriptional regulatory networks.

Certainly, our work can further be improved from the following aspects. First, we construct cell line specific regulatory network based on multiple tissues and cell lines, and how to construct sample-specific regulatory networks is also very important [55–57]. Second, we only consider regulatory relationships between transcription factors and target genes in the current work. It is known that DNA regulatory elements are of great importance in gene regulation. Therefore, the incorporation of regulatory elements into a regulatory network is necessary [58]. Third, there have been great innovations in experimental technology for studying the epigenome in the recent years. For example, ATAC-seq [59] has been proposed as an more efficient alternative of DNase-seq. HiChIP [60] has been developed to directly assess enhancer activity and enhancer-promoter interactions. These techniques have provided a great opportunity to study gene regulatory networks towards the understanding of phenotypic consequences of human genetic variation on physiology traits or disease risks. How to bring the idea of integrative analysis in our work to facilitate deep analysis regarding multiple types of epigenomic and transcriptomic data will be a direction worth noting.

## Additional file

Additional file 1: **Figure S1.** The directed acyclic subgraph of the human cell hierarchical taxonomy graph. **Figure S2** The similarity of Th2 cell and Crohn’s disease. **Figure S3** The enrichment degree for target set of NFKB1 in 110 cell lines. **Table S1** TFs and corresponding GO terms that alter between normal and cancer cell lines. **TableS2** Top ranked TFs based on their differential regulating edges. (DOC 501 kb)
