# Supplementary material for: Constructing tissue-specific transcriptional regulatory networks via a Markov random field
Source: BMC Genomics. 2018 Dec 31;19(Suppl 10):884. doi: 10.1186/s12864-018-5277-6 (PMC6311931; doi:10.1186/s12864-018-5277-6)
Supplement: Supplementary file 1 — Figure S1. The directed acyclic subgraph of the human cell hierarchical taxonomy graph. Figure S2 The similarity of Th2 cell and Crohn’s disease. Figure S3 The enrichment degree for target set of NFKB1 in 110 cell lines. Table S1 TFs and corresponding GO terms that alter between normal and cancer cell lines. TableS2 Top ranked TFs based on their differential regulating edges. (DOC 501 kb) [file 12864_2018_5277_MOESM1_ESM.doc]

# Constructing tissue-specific transcriptional regulatory networks via a Markov random field

**— Supplementary Materials**

### Shining Ma^1^, Tao Jiang^2,3,§^, and Rui Jiang^2,§^

^1^ Department of Statistics, Department of Biomedical Data Science, Bio-X Program Stanford University, Stanford, CA 94305, USA,

^2^ Ministry of Education Key Laboratory of Bioinformatics and Bio-informatics Division, Tsinghua National Laboratory for Information Science and Technology, Department of Automation, Tsinghua University, Beijing 100084, China,

^3^ Department of Computer Science and Engineering, University of California, Riverside, CA 92521, USA

^§^ Corresponding author

Email addresses:

SM: [snma@stanford.edu](mailto:snma@stanford.edu)

TJ: [jiang@cs.ucr.edu](mailto:jiang@cs.ucr.edu)

RJ: [ruijiang@tsinghua.edu.cn](mailto:ruijiang@tsinghua.edu.cn)

## Contents

**Supplementary Figures 2**

S1. The directed acyclic subgraph of the human cell hierarchical taxonomy graph 2

S2. The similarity of Th2 cell and Crohn’s disease 3

S3. The enrichment degree for target set of NFKB1 in 110 cell lines 4

**Supplementary Tables 5**

S1. TFs and corresponding GO terms that alter between normal and cancer cell lines 5

S2. Top ranked TFs based on their differential regulating edges 6

# Supplementary Figures


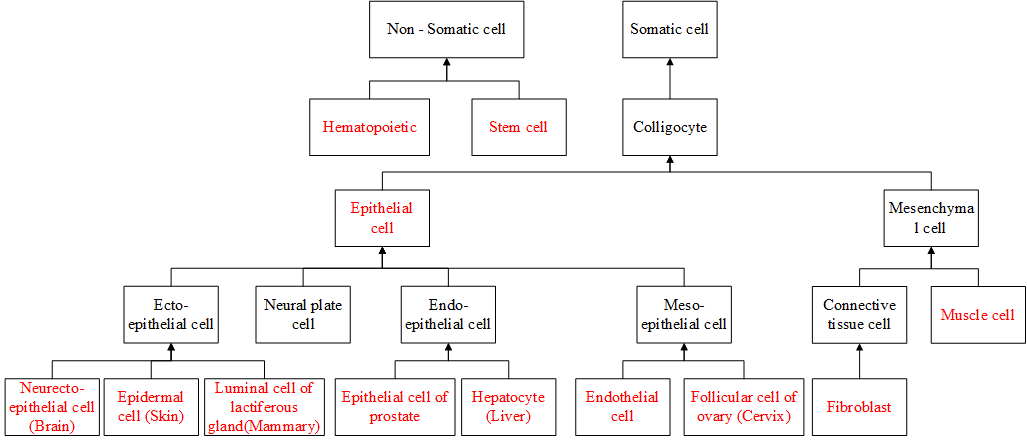


**Figure S1.** The directed acyclic subgraph of the human cell hierarchical taxonomy graph from Foundational Model of Anatomy Database in the Unified Medical Language System

**
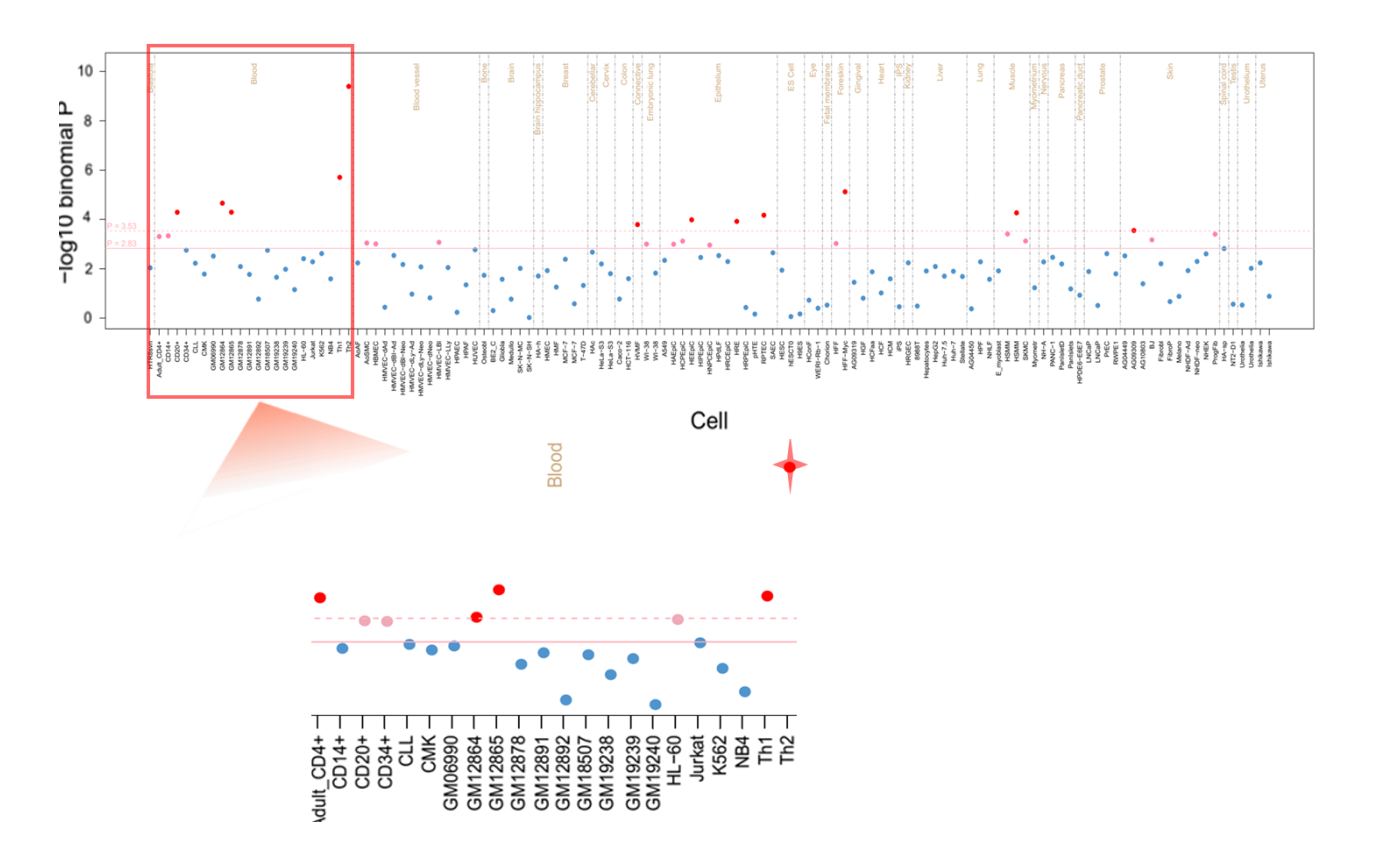
**

**Figure S2.** From 1000 Genomes Project, the similarity of Th2 cell and Crohn’s disease is observed to be the highest among the ENCODE cell lines.


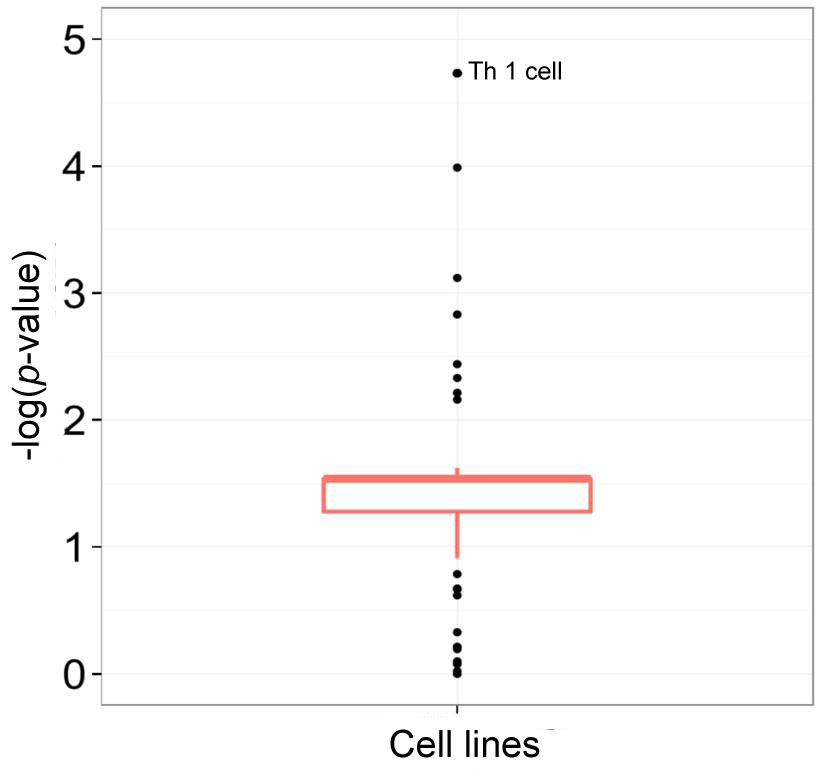


**Figure S3.** The enrichment degree for target set of NFKB1 in 110 cell lines, of which Th1 cell line is ranked 1^st^.

**Table S1**. TFs and corresponding GO terms that alter between normal and cancer cell lines

| **TF** | **GO ID** | **GO term** | ***p*-value** | **Pearson's Correlation** |
| --- | --- | --- | --- | --- |
| NFKB1 | GO:0006950 | response to stress | 0 | 0.778 |
| EP300 | GO:0006915 | apoptotic process | 0 | 0.754 |
|  | GO:0012501 | programmed cell death | 0 | 0.754 |
| FOSL1 | GO:0008283 | cell proliferation | 0 | 0.754 |
| EPAS1 | GO:0048518 | positive regulation of biological process | 0 | 0.774 |
|  | GO:0048522 | positive regulation of cellular process | 0 | 0.774 |
|  | GO:0045941 | positive regulation of transcription | 5.77E-15 | 0.709 |
|  | GO:0045893 | positive regulation of transcription, DNA-templated | 1.78E-14 | 0.699 |
| SMAD3 | GO:0016481 | negative regulation of transcription | 0 | 0.740 |
|  | GO:0016070 | RNA metabolic process | 1.33E-15 | 0.719 |
|  | GO:0031323 | regulation of cellular metabolic process | 5.77E-15 | 0.709 |
|  | GO:0045934 | negative regulation of nucleobase-containing compound metabolic process | 1.84E-14 | 0.699 |
|  | GO:0051253 | negative regulation of RNA metabolic process | 1.84E-14 | 0.699 |
|  | GO:0045892 | negative regulation of transcription, DNA-templated | 1.84E-14 | 0.699 |
|  | GO:0051169 | nuclear transport | 4.35E-14 | 0.692 |
|  | GO:0050790 | regulation of catalytic activity | 4.35E-14 | 0.692 |
|  | GO:0006913 | nucleocytoplasmic transport | 4.35E-14 | 0.692 |
|  | GO:0048519 | negative regulation of biological process | 6.59E-13 | 0.668 |
| TFAP4 | GO:0006351 | transcription, DNA-templated | 4.44E-16 | 0.727 |
|  | GO:0043283 | biopolymer metabolic process | 5.33E-15 | 0.709 |
| MXI1 | GO:0003677 | DNA binding | 1.23E-13 | 0.683 |
| REST | GO:0016070 | RNA metabolic process | 3.62E-14 | 0.693 |
|  | GO:0006351 | transcription, DNA-templated | 2.13E-13 | 0.678 |
|  | GO:0032774 | RNA biosynthetic process | 2.13E-13 | 0.678 |
|  | GO:0006350 | transcription | 2.13E-13 | 0.678 |
| NKX3-2 | GO:0016070 | RNA metabolic process | 1.84E-14 | 0.699 |
|  | GO:0043283 | biopolymer metabolic process | 1.81E-13 | 0.679 |
|  | GO:0006139 | nucleobase-containing compound metabolic process | 7.44E-13 | 0.667 |
| MYC | GO:0045449 | regulation of transcription | 2.47E-13 | 0.677 |

**Table S2**. Top ranked TFs based on their differential regulating edges

| **TF** | **Jaccard distance** | **No. of differentially regulating edges** | **Rank** |
| --- | --- | --- | --- |
| ING4 | 0.891534 | 12995 | 1 |
| NFATC2 | 0.98004 | 6825 | 2 |
| **SREBF1** | **0.808955** | **6504** | **3** |
| **TWIST** | **0.87782** | **5331** | **4** |
| ETS2 | 0.57934 | 4969 | 5 |
